# Hypoxia metabolism in ageing

**DOI:** 10.18632/aging.100782

**Published:** 2015-07-16

**Authors:** Alessandro Valli, Adrian L Harris, Benedikt M Kessler

**Affiliations:** Weatherall Institute of Molecular Medicine, University of Oxford; Target Discovery Institute, Nuffield Department of Medicine, University of Oxford, Oxford, UK

Ageing is characterized by a general decrease in O_2_ supply to tissues and a reduction in tissue pO_2_. A diminished vascularization in ageing alters the diffusion of O_2_ at the capillary tissue level, and at an advanced stage, this can lead to tissue hypoxia. Molecular O_2_ sensors mediate the response to O_2_ deprivation through the regulation of protein expression, enzyme activities, and metabolic regulating factors [1]. HIF1α is perhaps the best-studied responsive mechanism. A critical role in vascularization and angiogenesis is played by VEGF, a HIF1α target. This crucial proangiogenic factor is also regulated at the mRNA level by a non-HIF pathway, DEAD-box RNA helicase [2]. Deficient O_2_ supply together with a reduced ability to induce HIF1α expression may contribute to the aetiology of ageing. On the other hand, low O_2_ levels lead to neovascularization that can contribute to pathological events such as tumour growth and macular oedema. Furthermore, intermittent or continuous cellular hypoxia represents a source for oxygen radical production that is responsible for the inexorable decline of tissue morphology and physiology with age. In ageing, neovascularization appears to be attenuated which might be linked to impaired HIF1α induction. Other compensatory mechanisms might also take place in conditions of O_2_ limitation [1].

Our recent study made use of an integrated model of a human colorectal cancer cell line and genetically identical cell line lacking HIF1α expression, in a hypoxic environment [3]. This allowed the distinction between HIF1α-dependent and HIF1α-independent metabolic pathways, in particular for lipid metabolism. We observed that a number of lipid metabolic steps had a HIF1α-independent regulation in hypoxia, a trait that may have implications also in the ageing process. HIF2α expression and induction was independent of HIF1α [3].

HIF2α is associated with impaired fatty acid (FA) β-oxidation, increased cellular lipid storage, and has a pro-proliferative effect on cells, which can be an advantage for tumor growth. In well-oxygenated cells, FA β-oxidation represents an important source of energy that can account for up to 80% of supply in cardiomyocytes.

In our results, FA-synthase did not show up-regulation in hypoxia, indicating that O_2_ limitation did not induce the *de novo* FA biosynthesis [3]. This was in accordance with previous data reporting hypoxic cancer cells scavenging lipids from the extracellular environment to form lipid droplets [4]. In ageing, hypoxic oxidative stress causes peroxidation that transforms FA into hydroperoxide derivatives. These biochemical reactive species represent one of the major players in the free radical-mediated injury causing disease. We found palmitate and stearate, two saturated FAs, accumulating in HCT116 hypoxia cells in a HIF1α-independent manner [3]. FA are taken-up by cells through a passive “flip-flop” diffusion in which an FA ionization switch determines the kinetic rate of uptake. Alternatively, FA translocase-transport proteins and plasma membrane associated FA-binding proteins can transport FA into the cells [4]. We found hypoxia inducing an intracellular accumulation in excess of both saturated and unsaturated FA in cancer cells [3]. Such increase can provide metabolic precursors for recovering proliferating cells after re-oxygenation and act as a free radical buffer [4].

Our result showed that the platelet-activating factor (PAF) homologous C16 accumulated in hypoxia in a HIF1α/HIF2α-independent manner, an effect observed in multiple cell lines [3]. PAF is a potent, inflammatory lipid mediator that increases vascular permeability and vasodilatation, which may be related to its ability to stimulate the release of agents such as nitric oxide (NO). PAF, indeed, was found to evoke a rapid increase of total NO in human endometrial epithelial cells. PAF, which is one of the key factors relevant for atherogenesis, was suggested as a marker for atherosclerosis risk [5]. Our data suggest that the HIF-independent hypoxic accumulation of PAFC16, in HCT116 cells, could be due to a reduction of PAFC16 catabolism. This could be the result of a decrease in acetyl hydrolase and phospholipase D levels and/or O_2_-independent activity [3, 6]. Platelet activating factor receptor (PAFR) was found particularly active in tumours via the modulation of oncogenic transformation and angiogenesis. For instance, in ovarian cancer PAFR activation led to enhanced cell proliferation, invasion and tumour growth [6]. Many different cell types, including endothelial, stromal and inflammatory cells, secrete PAFR-PAF ligand, which is also secreted by many different tumour cells. PAF induced metabolism could therefore represent a significant metabolic pathway in hypoxia induced cancer metabolism, which might be also of interest in the ageing field [3, 5].

**Figure 1:**
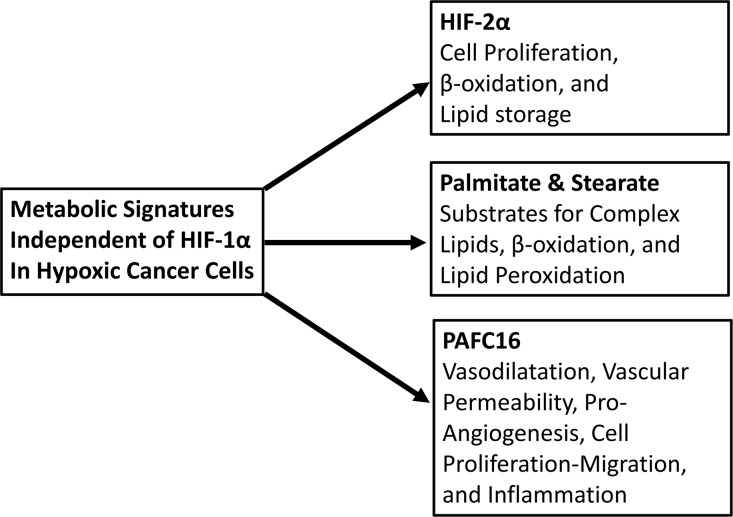
Metabolism mediated by HIF-1α-independent hypoxia. Similar metabolic alterations may occur in advanced senescence and aging. Scheme shows the detected features in HCT116 regulated in HIF-1α-independent manner after 24 hours of 1% oxygen cultivation [3].

Our study showed the prominent role of hypoxia in lipid metabolism [3]. The biological model utilized allows uncovering of hypoxic metabolic mechanisms that are regulated by O_2-_requiring enzymes, dioxygenases [7], which although dependent on O_2_, are independent of HIF1 or HIF2. The finding generated by our work can be translated in other fields of investigation, such as ageing, where hypoxia could play a major role in the regulation of ageing-associated diseases (Figure 1).
